# Tuning Surface Structure of Pd_3_Pb/Pt*_n_*Pb Nanocrystals for Boosting the Methanol Oxidation Reaction

**DOI:** 10.1002/advs.201902249

**Published:** 2019-10-29

**Authors:** Xingqiao Wu, Yi Jiang, Yucong Yan, Xiao Li, Sai Luo, Jingbo Huang, Junjie Li, Rong Shen, Deren Yang, Hui Zhang

**Affiliations:** ^1^ State Key Laboratory of Silicon Materials and School of Materials Science & Engineering Zhejiang University Hangzhou 310027 China

**Keywords:** bifunctional mechanism, electrocatalysis, intermetallics, platinum, surface engineering

## Abstract

Developing an efficient Pt‐based electrocatalyst with well‐defined structures for the methanol oxidation reaction (MOR) is critical, however, still remains a challenge. Here, a one‐pot approach is reported for the synthesis of Pd_3_Pb/Pt*_n_*Pb nanocubes with tunable Pt composition varying from 3.50 to 2.37 and 2.07, serving as electrocatalysts toward MOR. Their MOR activities increase in a sequence of Pd_3_Pb/Pt_3.50_Pb << Pd_3_Pb/Pt_2.07_Pb < Pd_3_Pb/Pt_2.37_Pb, which are substantially higher than that of commercial Pt/C. Specifically, Pd_3_Pb/Pt_2.37_Pb electrocatalysts achieve the highest specific (13.68 mA cm^−2^) and mass (8.40 A mg_Pt_^−1^) activities, which are ≈8.8 and 6.8 times higher than those of commercial Pt/C, respectively. Structure characterizations show that Pd_3_Pb/Pt_2.37_Pb and Pd_3_Pb/Pt_2.07_Pb are dominated by hexagonal‐structured PtPb intermetallic phase on the surface, while the surface of Pd_3_Pb/Pt_3.50_Pb is mainly composed of face‐centered cubic (fcc)‐structured Pt*_x_*Pb phase. As such, hexagonal‐structured PtPb phase is much more active than the fcc‐structured Pt*_x_*Pb one toward MOR. This demonstration is supported by density functional theory calculations, where the hexagonal‐structured PtPb phase shows the lowest adsorption energy of CO. The decrease in CO adsorption energy and structural stability also endows Pd_3_Pb/Pt*_n_*Pb electrocatalysts with superior durability relative to commercial Pt/C.

## Graphical abstract

Pd_3_Pb/Pt*_n_*Pb nanocubes with tunable surface composition and structure are synthesized by a one‐pot approach. Hexagonal‐structured PtPb intermetallics on the surface and the incorporation of Pd_3_Pb cores endow Pd_3_Pb/Pt_2.37_Pb electrocatalysts with remarkably enhanced activity and durability toward the methanol oxidation reaction relative to commercial Pt/C due to the lower CO absorption energy.

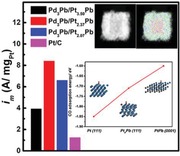

In the past decades, overuse of nonrenewable fossil fuel brought human society some serious issues including environment pollution and energy crisis, encouraging scientific researchers to develop a clean and sustainable energy source.^1^, ^2^ Direct methanol fuel cells (DMFCs) have been considered as one of the most promising candidates for portable and mobile applications due to their high conversion efficiency, low pollutant emission, and relative convenience to handle the liquid fuel.^3^, ^4^, ^5^, ^6^ Compared to the electro‐oxidation of hydrogen, however, methanol oxidation reaction (MOR) contains multiple steps, resulting in much slower reaction kinetics and thus compromising the overall performance of DMFCs.^7^, ^8^, ^9^, ^10^ As is well known, Pt is a key component of the electrocatalysts for MOR in DMFCs, which is severely restricted by its scarce abundance and high cost in practical applications.^11^, ^12^, ^13^, ^14^ In addition, the electrocatalysts consisting of pure Pt are extremely vulnerable to the poisoning effect of intermediates such as CO produced during MOR.^15^, ^16^, ^17^, ^18^ As such, developing a low‐cost, highly active, and stable Pt‐based electrocatalyst toward MOR is the most urgent request for widespread commercialization of DMFCs.

To meet the requirement for their commercial applications, tremendous efforts have been devoted to tuning Pt‐based nanocrystals by various strategies, including composition optimization, shape control, structure design, and so on.^19^, ^20^, ^21^, ^22^ Among them, alloying Pt with oxophilic metals, such as Ru, Ni, Cu, and so forth, is a commonly used strategy to improve the MOR properties by enhancing the tolerance of Pt toward CO poisoning according to the bifunctional mechanism and/or ligand effect.^23^, ^24^, ^25^, ^26^, ^27^ In addition, introduction of second metals into the electrocatalysts is expected to improve the utilization efficiency of Pt. Recently, DMFCs operating in an alkaline media have received abundant research interests due to some additional advantages such as improved reaction kinetics and less corrosive environment to the electrocatalysts.^28^, ^29^, ^30^ However, Pt is not sufficiently active in water dissociation and thus highly susceptible to poisoning by surface‐adsorbed reaction intermediates such as CO. Metal hydroxide such as Ni(OH)_2_ is usually employed to promote the dissociative adsorption of water molecules and subsequent oxidative removal of carbonaceous intermediates, leading to enhancement in MOR properties in terms of activity and stability.^15^, ^31^ Although significant advances have been achieved in the synthesis of efficient Pt‐based electrocatalysts for MOR, their MOR properties still have a large room for further improvement.

Compared to Pt‐based random alloys, ordered intermetallic compounds with well‐defined stoichiometry and crystal structure facilitate predictable control of structural, electronic, and geometric effects, which have received great attention in the field of advanced catalysts.^32^, ^33^, ^34^, ^35^, ^36^ Due to the long‐range ordered structure and more negative enthalpy of formation, intermetallic nanocrystals exhibit superior catalytic activity and stability relative to disordered counterparts.^37^ For instance, Murray and co‐workers have successfully synthesized intermetallic Pt_3_Zn nanoparticles, which exhibited greater CO tolerance and higher activity toward MOR compared to their random alloys.^38^ In another study, Li and co‐workers reported the synthesis of a tensile‐strained two‐to‐three atomic‐layer Pt on intermetallic Pt_3_Ga nanoparticles, exhibiting substantially enhanced MOR properties relative to commercial Pt/C.^39^ Recently, massive efforts have been employed to synthesize PtPb related intermetallic nanocrystals as electrocatalysts for MOR.^40^, ^41^ However, it still remains a challenge to tune surface composition of Pt*_n_*Pb intermetallic nanocrystals for facilitating MOR, especially those with well‐defined shape.

Here, we report one‐pot approach for synthesizing Pd_3_Pb/Pt*_n_*Pb nanocubes with ordered surface and tunable Pt compositions. Such Pd_3_Pb/Pt*_n_*Pb nanocubes exhibited substantially enhanced catalytic properties for MOR relative to commercial Pt/C. Of them, Pd_3_Pb/Pt_2.37_Pb nanocubes achieved the highest activity and stability toward MOR due to the lowest adsorption strength of CO on their surface.

The Pd_3_Pb/Pt*_n_*Pb nanocrystals were prepared by reduction of Pd(acac)_2_, Pb(acac)_2_, and H_2_PtCl_6_·*x*H_2_O in oleylamine (OAm) at 180 °C. **Figure** **1** shows morphological, structural, and compositional characterizations of the Pd_3_Pb/Pt*_n_*Pb nanocrystals that were obtained by adding 10.5 mg of Pb(acac)_2_ (defined as the standard procedure). From the transmission electron microscopy (TEM) image in Figure 1a, most of the nanocrystals have a uniform cube‐like morphology with average edge length of ≈50 nm. In addition, the surface of the nanocubes are rough and composed of various small nanoparticles (Figure S1, Supporting Information), indicating polycrystalline structure. This demonstration was supported by the selected area electron diffraction (SAED) pattern with a ring‐like feature (Figure 1b). After measuring the radius of the diffraction rings, the red dotted semicircles belong to PtPb phase with a hexagonal structure, and the blue and black ones possibly correspond to Pt*_x_*Pb and/or Pd_3_Pb phases with a face‐centered cubic (fcc) structure since these two phases have similar diffraction patterns. To determine the phase structure of the nanocubes, the X‐ray diffraction (XRD) technique was employed, as shown in Figure 1c. As observed, the peaks at 38.8°, 45.1°, 65.5°, and 78.7° can be attributed to fcc‐structured Pt*_x_*Pb (JCPDS 06‐0574) and/or Pd_3_Pb (JCPDS 50‐1631) phases. It is still difficult to differentiate these two phases by XRD analysis. The remaining peaks can be indexed to hexagonal‐structured PtPb (JCPDS 06‐0374) phase, which is consistent with the SAED result. In addition, the elemental distribution of Pd, Pt, and Pb in the nanocubes was determined by energy dispersive X‐ray (EDX) mapping and line‐scan analyses, as shown in Figure 1d,e. Clearly, Pd is concentrated in the interior, Pt only exists on the surface, while Pb is distributed throughout the nanocubes. Combined with the XRD result, the nanocubes are composed of Pd_3_Pb phase in the interior. To characterize the fine structure on the surface of the nanocubes, the aberration‐corrected high‐angle annular dark‐field scanning TEM (HAADF‐STEM) technique was further employed. As shown in Figure 1f, there are two kinds of lattice fringes with a spacing of 0.24 and 0.21 nm along the tip (marked by red arrows), corresponding to the (111) facet of Pt*_x_*Pb phase and the (110) facet of PtPb phase, respectively. In addition, an interface between Pt*_x_*Pb and PtPb can be clearly observed, which is marked by red dots (see Figure S2 in the Supporting Information for how to determine the interface region). This demonstration was supported by the HAADF‐STEM images (Figure S3, Supporting Information) taken from other region. Taken together, the nanocubes are composed of fcc‐structured Pd_3_Pb intermetallics, fcc‐structured Pt*_x_*Pb, and hexagonal‐structured PtPb intermetallics from inside to outside. The atomic ratio of Pt/Pb was about 2.37 as quantitatively determined by inductively coupled plasma atomic emission spectrometry (ICP‐AES, Table S1, Supporting Information). This sample was labeled as Pd_3_Pb/Pt_2.37_Pb for simplicity.

**Figure 1:**
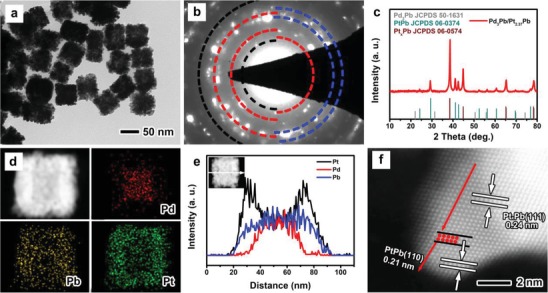
Morphological, structural, and compositional characterizations of the Pd_3_Pb/Pt_2.37_Pb nanocubes prepared using the standard procedure: a) TEM image, b) SAED pattern, c) XRD pattern, d) EDX mapping image, e) line‐scan profiles, and f) aberration‐corrected HAADF‐STEM image.

The surface composition of the nanocubes was tuned by varying the amount of Pb precursor in the syntheses. When more Pb(acac)_2_ (12 mg) or less Pb(acac)_2_ (7 mg) was added, both the samples showed a cubic‐like shape with rough surface (Figures S4a and S5a, Supporting Information). Similar to the Pd_3_Pb/Pt_2.37_Pb nanocubes, they are polycrystalline in nature (Figures S4b and S5b, Supporting Information). From the XRD (Figures S4c and S5c, Supporting Information), high‐resolution TEM (HRTEM; Figures S4d and S5d, Supporting Information), HAADF‐STEM‐EDX mapping (Figures S4e and S5e, Supporting Information), and line‐scan (Figures S4f and S5f, Supporting Information) analyses, the sample prepared by adding 12 mg of Pb(acac)_2_ is also composed of fcc‐structured Pd_3_Pb intermetallics, fcc‐structured Pt*_x_*Pb, and hexagonal‐structured PtPb intermetallics from inside to outside. ICP‐AES analysis shows that the atomic ratio of Pt/Pb in this sample is about 2.07, which is labeled as Pd_3_Pb/Pt_2.07_Pb (Table S1, Supporting Information). When 7 mg of Pb(acac)_2_ was fed in the synthesis, the structure of the sample is similar except for the absence of hexagonal‐structured PtPb phase (Figure S5c,d, Supporting Information). The atomic ratio of Pt/Pb was measured to be 3.50 (labeled as Pd_3_Pb/Pt_3.50_Pb).

In order to clarify the growth mechanism of the Pd_3_Pb/Pt*_n_*Pb nanocubes, a series of samples were taken from the reacting solution at different reaction time for TEM (Figure S6, Supporting Information) and XRD (Figure S7, Supporting Information) characterizations. Here, the Pd_3_Pb/Pt_2.37_Pb nanocubes were selected as a typical example for monitoring the formation process. In the initial stage of the reaction (Figure S6a, Supporting Information; *t* = 10 min), some nanocubes with average edge length of ≈10 nm coexisted with a rich variety of small aggregated nanoparticles. The XRD analysis (Figure S7, Supporting Information) shows that the nanocubes are composed of fcc‐structured Pd_3_Pb and the aggregated nanoparticles are the intermediates associated with Pb^2+^, OH^−^, and Cl^−^ ions. When the reaction was extended to *t* = 15 min (Figure S6b, Supporting Information), more Pd_3_Pb nanocubes with the increased size were formed, accompanied by the decrease in the amount of the aggregated nanoparticles. This demonstration was confirmed by the XRD result with the increase in the peak intensity of Pd_3_Pb and the decrease in the peak intensity of Pb‐related intermediates. Such transformation was continued with extension of the reaction time (Figures S6c and S7, Supporting Information; *t* = 20 min). The HRTEM image (Figure S8a, Supporting Information) and EDX line‐scan spectra (Figure S8b, Supporting Information) indicate the formation of Pt*_x_*Pb phase on the surface of the nanocubes. When the reaction proceeded to 30 min, the nanocubes with rough surface were formed in combination with the disappearance of Pb related intermediates (Figure S6d, Supporting Information). Simultaneously, the hexagonal‐structured PtPb phase began to appear (Figure S7, Supporting Information). As such, the synthesis of the Pd_3_Pb/Pt*_n_*Pb nanocrystals involved the initial formation of Pd_3_Pb nanocubes and Pb related intermediates, the transformation of Pb related intermediates, and the interdiffusion of Pt and Pb atoms to form the Pt*_x_*Pb and PtPb phases in a sequence. In the absence of Pd(acac)_2_, irregular large particles instead of the nanocubes were generated, which were composed of Pt*_x_*Pb and PtPb phase (Figure S9, Supporting Information; labeled as Pt*_x_*Pb/PtPb), indicating that the initially formed Pd_3_Pb nanocubes serve as the template for the formation of the Pd_3_Pb/Pt*_n_*Pb nanocubes.

The surface composition and valence state of three Pd_3_Pb/Pt*_n_*Pb nanocubes were analyzed using the X‐ray photoelectron spectroscopy (XPS) technique. The Pt 4f and Pb 4f XPS spectra were calibrated with respect to the binding energies (BEs) of the C 1s peak of graphite at 284.5 eV, as shown in **Figure** **2**. As observed, Pt and Pb elements on the surface of the nanocubes existed in both zerovalent and divalent states for these three samples, with the metallic state being the majority. Compared with the BE of Pt^0^ in commercial Pt/C (Figure S10, Supporting Information) and bulk Pt (Table S2, Supporting Information), there is an obvious negative shift for the three nanocubes, with a shift value in a sequence of Pd_3_Pb/Pt_3.50_Pb (Figure 2a) < Pd_3_Pb/Pt_2.37_Pb (Figure 2c) < Pd_3_Pb/Pt_2.07_Pb (Figure 2e). For example, the BE of Pt^0^ in the Pd_3_Pb/Pt_2.37_Pb nanocubes located at 70.96 (4f_7/2_) and 74.29 (4f_5/2_) eV, which displayed a negative shift of 0.24 eV relative to those of bulk Pt. Noticeably, an opposite trend associated with Pb^0^ 4f_7/2_ and Pb^0^ 4f_5/2_ in these three nanocubes was observed compared with those of bulk Pb (Figure 2b,d,f). These results demonstrated that a strong electron coupling occurred between Pt and Pb (i.e., ligand effect) because of their different work function, which might facilitate MOR.^42^, ^43^, ^44^ In addition, the atomic ratios of Pt/Pb on the surface of the nanocubes were calculated from the XPS spectra, as shown in Table S2 in the Supporting Information. Clearly, the atomic ratios of Pt/Pb on the surface decreased with the increase in the amount of Pb precursor fed in the synthesis, indicating the increase in the amount of PtPb phase. This result is consistent with the HRTEM image and XRD data.

**Figure 2:**
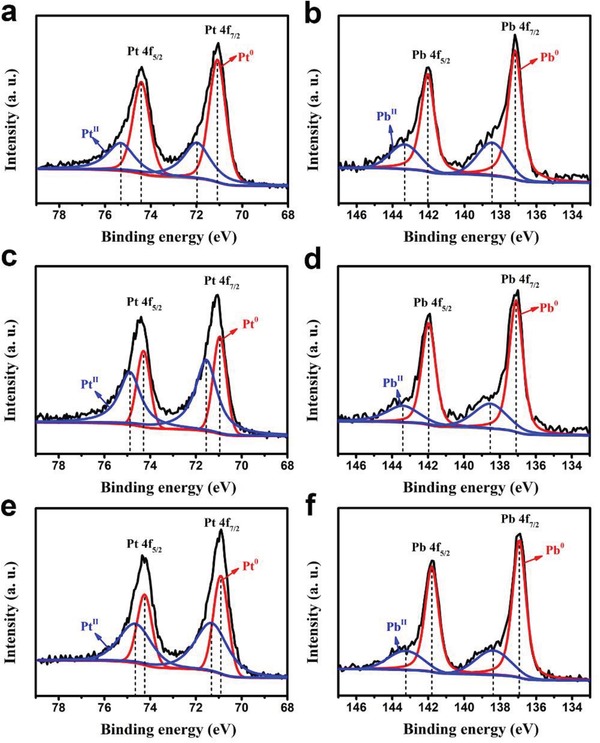
a,c,e) Pt 4f and b,d,f) Pb 4f XPS spectra of the Pd_3_Pb/Pt_3.50_Pb, Pd_3_Pb/Pt_2.37_Pb, and Pd_3_Pb/Pt_2.07_Pb nanocubes, respectively.

The Pd_3_Pb/Pt*_n_*Pb nanocubes were loaded on carbon support (Vulcan XC‐72R), and then evaluated as the electrocatalysts for MOR with commercial Pt/C as a reference. To clean the surface of these four carbon‐supported electrocatalysts, the cyclic voltammetry (CV) was first conducted at room temperature in Ar‐purged 1 m KOH solutions at a sweep rate of 50 mV s^−1^ between 0 and 1.0 V versus reversible hydrogen electrode (RHE) for 50 cycles. Their electrochemically active surface areas (ECSAs) were obtained by measuring the charge of CO oxidation in the CO stripping curves (Figure S11, Supporting Information). Compared to the commercial Pt/C (79.7 m^2^ g^−1^), the Pd_3_Pb/Pt*_n_*Pb nanocubes exhibited the relatively smaller ECSAs (61.4 m^2^ g^−1^ for Pd_3_Pb/Pt_3.50_Pb, 61.2 m^2^ g^−1^ for Pd_3_Pb/Pt_2.37_Pb, and 49.3 m^2^ g^−1^ for Pd_3_Pb/Pt_2.07_Pb) due to their larger particle sizes. **Figure** **3**a,b compares the CV curves of such four electrocatalysts for MOR recorded in an aqueous solution containing 1 m KOH and 1 m MeOH. As observed, the Pd_3_Pb/Pt*_n_*Pb nanocubes display the substantially enhanced specific and mass activities for MOR compared to the commercial Pt/C. Specifically, the Pd_3_Pb/Pt_2.37_Pb nanocubes achieved the highest specific (13.68 mA cm^−2^) and mass (8.40 A mg_Pt_^−1^) activities, which are ≈8.8 and 6.8 times higher than those of commercial Pt/C (Figure 3c). Table S3 in the Supporting Information summarizes the specific and mass activities of the Pd_3_Pb/Pt_2.37_Pb nanocubes in this work and state of art electrocatalysts in literatures. Clearly, the Pd_3_Pb/Pt_2.37_Pb nanocubes outperformed over most of the previously reported MOR electrocatalysts in alkaline media. To determine the effect of the Pd_3_Pb cores on the MOR properties of the Pd_3_Pb/Pt*_n_*Pb nanocubes, the Pd_3_Pb intermetallic nanocubes with average edge lengths of ≈50 nm were synthesized in OAm containing hexadecyl trimethyl ammonium chloride (CTAC) by co‐reducing Pd and Pb precursors (see the Supporting Information for the detail), as shown in Figure S12 in the Supporting Information. The catalytic measurement (Figure S13, Supporting Information) shows the negligible activity of the Pd_3_Pb nanocubes for MOR. This result indicates that Pt*_n_*Pb phase is the active component toward MOR. Moreover, the PtPb nanoparticles were synthesized in a mixed solution containing OAm and 1‐octadecene (ODE) in an aim to reveal the role of Pd_3_Pb core in MOR properties. The TEM image, HRTEM image, EDX line‐scan spectra, and XRD pattern indicate that the nanoparticles were composed of hexagonal PtPb intermetallic phase with Pt and Pb elements being distributed uniformly throughout the nanoparticle (Figure S14, Supporting Information). The comparison of XPS data between the PtPb nanoparticles and three Pd_3_Pb/Pt*_n_*Pb nanocubes (Figure S15 and Table S1, Supporting Information) shows that incorporating Pd into the particle leaded to obvious negative shift of BE of Pt^0^, implying a strong electron coupling between Pd and Pt in the Pd_3_Pb/Pt*_n_*P nanocubes. From Figure S16 in the Supporting Information, the PtPb nanoparticle exhibited a mass activity of 4.36 A mg_Pt_^−1^, which was much lower than that (8.40 A mg_Pt_^−1^) of the Pd_3_Pb/Pt_2.37_Pb nanocubes. This result indicates that the ligand effect between Pd and Pt was responsible for the enhancement in MOR performance.

**Figure 3:**
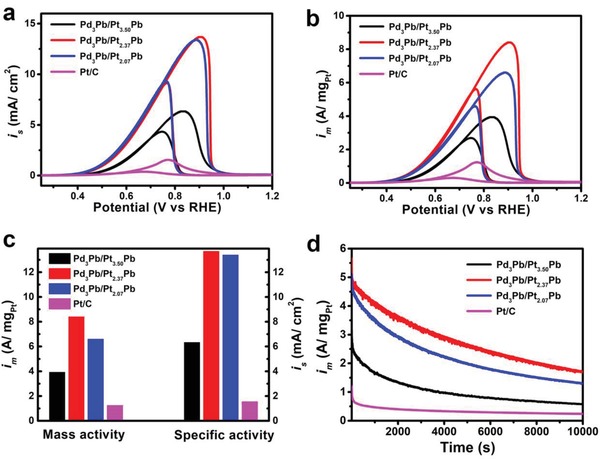
a,b) Cyclic voltammograms (CV) of the Pd_3_Pb/Pt_3.50_Pb nanocubes, Pd_3_Pb/Pt_2.37_Pb nanocubes, Pd_3_Pb/Pt_2.07_Pb nanocubes, and commercial Pt/C in a mixed solution containing 1 m KOH and 1 m MeOH at a scan rate of 50 mV s^−1^ for MOR normalized by surface area (CO stripping) and Pt mass, respectively. c) Mass and specific activities at the peak position of forward curve. d) Current–time (*I*–*t*) curves for MOR at 0.77 V (vs RHE) for 10 000 s.

In addition, the MOR activities of three Pd_3_Pb/Pt*_n_*Pb nanocubes varied with the surface composition of Pt in a sequence of Pd_3_Pb/Pt_2.37_Pb > Pd_3_Pb/Pt_2.07_Pb >> Pd_3_Pb/Pt_3.50_Pb (Figure 3c). However, the Pd_3_Pb/Pt_2.37_Pb and Pd_3_Pb/Pt_2.07_Pb nanocubes exhibited very similar specific activity for MOR (13.68 vs 13.39 mA cm^−2^), which is 2.1 times higher than that of the Pd_3_Pb/Pt_3.50_Pb nanocubes (6.33 mA cm^−2^). This result indicates that the Pd_3_Pb/Pt_2.37_Pb and Pd_3_Pb/Pt_2.07_Pb nanocubes are much more active intrinsically for MOR relative to the Pd_3_Pb/Pt_3.50_Pb nanocubes. Clearly, the difference in mass activity between the Pd_3_Pb/Pt_2.37_Pb and Pd_3_Pb/Pt_2.07_Pb nanocubes might result from their different ECSAs. Combined with compositional and structural characterizations, hexagonal‐structured PtPb phase might be more active for MOR than fcc‐structured Pt*_x_*Pb phase since PtPb phase with a hexagonal structure was the major component on the surface of both Pd_3_Pb/Pt_2.37_Pb and Pd_3_Pb/Pt_2.07_Pb nanocubes, while the surface of the Pd_3_Pb/Pt_3.50_Pb nanocubes was dominated by Pt*_x_*Pb phase with fcc structure.

The enhanced MOR activities of three Pd_3_Pb/Pt*_n_*Pb nanocubes can also be understood by well‐known bifunctional mechanism.^45^, ^46^ In general, Pt is the active site for methanol adsorption and dissociation during MOR, resulting in the formation of the intermediates mainly containing CO_ads_. Such CO_ads_ intermediates can severely poison these active sites of Pt through strong adsorption, and eventually deactivate Pt‐based electrocatalysts. The incorporation of oxophilic metal like Ru, Sn, and Pb can dissociate water to form the oxygenated species such as OH_ads_.^47^, ^48^, ^49^, ^50^, ^51^ Such oxygenated species can promote the further oxidation of CO_ads_, and then remove them from Pt sites, which substantially enhance the catalytic properties of these three Pd_3_Pb/Pt*_n_*Pb nanocubes toward MOR. As such, the adsorption energy of CO on Pt‐based electrocatalysts is a critical descriptor of the intrinsic activity for MOR.^52^, ^53^, ^54^, ^55^ In order to deeply understand the difference in MOR activity of three Pd_3_Pb/Pt*_n_*Pb nanocubes, the adsorption energies of CO on fcc‐structured Pt(111), fcc‐structured Pt*_x_*Pb(111), and hexagonal‐structured PtPb(0001) were obtained by the density functional theory (DFT) calculations. The theoretical models (**Figure** **4**; Figure S17, Supporting Information) were constructed for fcc‐structured Pt(111), fcc‐structured Pt*_x_*Pb(111), and hexagonal‐structured PtPb(0001) based on the corresponding structural and compositional parameters. As observed from Figure 4 and Table S4 in the Supporting Information, the adsorption energies of CO on these three phases followed a sequence of fcc‐structured Pt(111) > fcc‐structured Pt*_x_*Pb(111) > hexagonal‐structured PtPb(0001), indicating that hexagonal‐structured PtPb(0001) is the most active component for MOR among them. As such, the Pd_3_Pb/Pt_2.37_Pb and Pd_3_Pb/Pt_2.07_Pb nanocubes with hexagonal‐structured PtPb intermetallics on the surface show higher catalytic activities for MOR than the Pd_3_Pb/Pt_3.50_Pb nanocubes with fcc‐structured Pt*_x_*Pb as the main phase on the surface.

**Figure 4:**
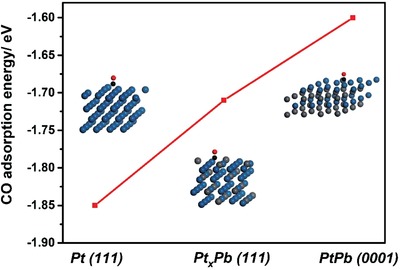
CO adsorption energy on three kinds of surfaces including fcc‐structured Pt(111), fcc‐structured Pt*_x_*Pb(111), and hexagonal‐structured PtPb(0001). Insets showing the structure models for above‐mentioned three surfaces with an adsorbed CO molecule. C, O, Pt, and Pb atoms were represented by black, red, blue, and gray balls.

At last, the stability of the above‐mentioned four electrocatalysts was investigated by long‐term current–time (*I*–*t*) measurement performed at 0.77 V for 10 000 s. As shown in Figure 3d, the three Pd_3_Pb/Pt*_n_*Pb nanocubes show the higher steady current density relative to commercial Pt/C for MOR over the entire time range (10 000 s), indicating their superior durability. Among them, the Pd_3_Pb/Pt_2.37_Pb nanocubes remained the highest mass activity (1.73 A mg_Pt_^−1^) after 10 000 s stability test, which is much larger than that of commercial Pt/C (0.24 A mg_Pt_^−1^). Such superior durability can be attributed to weaker CO adsorption on the Pd_3_Pb/Pt*_n_*Pb electrocatalysts and better CO tolerance due to the incorporation of oxophilic Pb through well‐known bifunctional mechanism. From the TEM images in Figure S18 in the Supporting Information, the Pd_3_Pb/Pt_2.37_Pb electrocatalysts are still well dispersed on carbon support after the electrochemical measurements, indicating that their structural stability is also responsible for the enhanced catalytic durability due to more negative formation enthalpy of the intermetallic compounds.

In summary, we have developed a facile one‐pot approach to synthesize the Pd_3_Pb/Pt*_n_*Pb nanocubes with different Pt compositions (*n* = 3.50, 2.37, 2.07). Such tunable composition led to the difference in surface structure of the Pd_3_Pb/Pt*_n_*Pb nanocubes, i.e., hexagonal‐structured PtPb intermetallics were the major phase in both the Pd_3_Pb/Pt_2.37_Pb and Pd_3_Pb/Pt_2.07_Pb, while the surface of the Pd_3_Pb/Pt_3.50_Pb was dominated by fcc‐structured Pt*_x_*Pb phase. The Pd_3_Pb/Pt*_n_*Pb nanocubes exhibited substantially enhanced activity and durability toward MOR relative to commercial Pt/C and PtPb intermetallic nanoparticles, with the Pd_3_Pb/Pt_2.37_Pb being the best MOR electrocatalysts and outperforming over most of the previously reported Pt‐based ones. DFT calculations show that the PtPb phase is more active component for MOR relative to the Pt*_x_*Pb and Pt phases due to the increased adsorption energies of CO on them in a sequence of PtPb < Pt*_x_*Pb < Pt. The incorporation of Pd_3_Pb core was also responsible for the enhancement in MOR activities through a ligand effect. In addition, the Pd_3_Pb/Pt*_n_*Pb nanocubes were more stable toward MOR than commercial Pt/C due to their better CO tolerance and unique intermetallic surface structure with higher chemical stability. This work not only provides a facile method to the synthesis of Pt‐based nanocubes with ordered surface, but also opens up new opportunities to design advanced Pt‐based MOR electrocatalysts by surface engineering.

## Conflict of Interest

The authors declare no conflict of interest.

## Supporting information

Supporting Information
