# Supplementary material for: Tuning Surface Structure of Pd3Pb/PtnPb Nanocrystals for Boosting the Methanol Oxidation Reaction
Source: Adv Sci (Weinh). 2019 Oct 29;6(24):1902249. doi: 10.1002/advs.201902249 (PMC6918111; doi:10.1002/advs.201902249)
Supplement: Supplementary file 1 — Supporting Information [file ADVS-6-1902249-s001.pdf]

Copyright WILEY-VCH Verlag GmbH & Co. KGaA, 69469 Weinheim, Germany, 2019.

ADVANCED SCIENCE
Open Access

Supporting Information

for *Adv. Sci.,* DOI: 10.1002/advs.201902249

Tuning Surface Structure of Pd_3Pb/Pt_nPb Nanocrystals for Boosting the Methanol Oxidation Reaction

*Xingqiao Wu, Yi Jiang, Yucong Yan, Xiao Li, Sai Luo, Jingbo Huang, Junjie Li, Rong Shen, Deren Yang, and Hui Zhang**

Supporting Information

Tuning Surface Structure of Pd_3Pb/Pt_nPb Intermetallic Nanocrystals for Boosting Methanol Oxidation Reaction

Xingqiao Wu, Yi Jiang, Yucong Yan, Xiao Li, Sai Luo, Jingbo Huang, Junjie Li, Rong Shen, Deren Yang, and Hui Zhang*

DOI:

Contents of Supporting Information

Materials and Methods

Supporting Tables S1-S4

Supporting Figures S1-S18

References

Materials and Methods

Chemical and Materials.

Palladium(II) acetylacetonate (Pd(acac)$_2$, 99%), lead(II) acetylacetonate (Pb(acac)$_2$, technical grade), chloroplatinic(IV) acid ($H_2PtCl_6 \cdot xH_2O$, 99.9%), hexadecyl trimethyl ammonium chloride (CTAC, 98%), n-butylamine (98%), 1-octadecene (ODE, 90%), potassium hydroxide (KOH, 99.99%) and methanol anhydrous (MeOH, 99.8%) were all purchased from Sigma Aldrich. Commercial Pt/C (20 wt%) were purchased from Alfa Aesar. Oleylamine (OAm, 80%-90%) was purchased from Aladdin. Ethanol, cyclohexane, chloroform and sulfuric acid (H_2SO_4, 98%) were purchased from Sinopharm Chemical Reagent. All the chemicals and materials were used as received. All aqueous solutions were prepared using ultrapure water with a resistivity of 18.2 MΩ·cm.

Synthesis of Pd_3Pb/Pt_nPb nanocubes.

In a standard procedure for synthesis of $Pd_3Pb/Pt_{2.37}Pb$, 3.0 mg of Pd(acac)$_2$, 10.5 mg of Pb(acac)$_2$, and 20.5 mg of $H_2PtCl_6 \cdot xH_2O$ were dissolved in a 6 mL of OAm and stirred for 2 h at room temperature. The homogeneous solution was then transferred into a 20 mL vial. The sealed vial was heated at 180 °C under magnet stirring for 1 h and then cooled to room temperature. The product was collected by centrifugation, washed with cyclohexane and ethanol for three times. For the synthesis of $Pd_3Pb/Pt_{3.50}Pb$ and $Pd_3Pb/Pt_{2.07}Pb$ nanocubes, the amount of Pb(acac)$_2$ was adjusted to 7 and 12 mg, respectively, while keeping other conditions unchanged. For the synthesis of $Pt_xPb/PtPb$ nanoparticles, only Pd(acac)$_2$ is absence with other conditions being the same as the standard procedure.

Synthesis of Pd_3Pb intermetallic nanocubes

In a typical synthesis, 9.1 mg of Pd(acac)$_2$, 4.05 mg of Pb(acac)$_2$, and 60 mg of CTAC were dissolved in a mixed solution of 2.5 mL OAm and 2.5 ml ODE, and then stirred for 2 h at room temperature. The homogeneous solution was then transferred into a 20 mL vial. The sealed vial was heated at 180 °C under magnet stirring for 3 h and then cooled to room temperature. The product was collected by centrifugation, washed with cyclohexane and ethanol for three times.

Synthesis of PtPb intermetallic nanoparticles

The PtPb intermetallic nanoparticles were synthesized according to the previous report.[1] In a typical synthesis, 12.0 mg of Pt(acac)$_2$, 8.0 mg of Pb(acac)$_2$, 4.5 mg of L-ascorbic acid, 33 mg of phenol, 2.5 mL of OAm and 2.5 mL of ODE were added into a 20 mL glass vial. After the vial had been capped, the mixture was ultrasonicated for 60 min. The resulting homogeneous mixture was then heated from room temperature to 160 °C in 30 min and maintained at 160 °C for 5 h in an oil bath, before it cooled to room temperature. The resulting colloidal products were collected by centrifugation and washed three times with an ethanol/cyclohexane mixture.

Morphological and structural characterizations.

Transmission electron microscopy (TEM) images of the obtained samples were taken using a HITACHI HT-7700 microscope operated at 100 kV. High-resolution transmission electron microscopy (HRTEM) and energy dispersive X-ray (EDX) analyses were performed using a FEI Tecnai F20 G2 microscope operated at 300 kV. Aberration-corrected high-angle annular dark-field scanning TEM (HAADF-STEM) was taken on a FEI Titan ChemiSTEM equipped with a probe-corrector and a Super-X EDX detector system. This microscope was operated at 200 kV with a probe current of 50 pA and a convergent angle of 21.4 mrads for illumination. The X-ray diffraction (XRD) patterns were recorded on a Miniflex600 X-ray diffractometer in a scan range of 10-80° at a scan rate of 10°/min. X-ray photoelectron spectrometer (XPS) was performed on ESCALAB 250Xi (Thermo, U.K). The corresponding binding energies were calibrated with a C-C 1s peak of 284.5 eV. The percentages of Pd, Pt, and Cu in the samples were determined using inductively coupled plasma atomic emission spectrometry (ICP-AES, IRIS Intrepid II XSP, TJA Co., USA).

Details in Electrochemical Measurements.

The electrochemical performances of the catalysts including $Pd_3Pb/Pt_nPb/C$, Pd_3Pb/C, $Pt_xPb/PtPb/C$, PtPb/C and commercial Pt/C were measured by a three-electrode cell using a CHI760E electrochemical analyser with a glassy-carbon rotating disk electrode (RDE, area: ~0.196 cm^2), a Pt mesh (1×1 cm^2), and a saturated calomel electrode (SCE) as the working, counter, and reference electrode, respectively. The as-received data were finally converted to reversible hydrogen electrode (RHE) as the reference. To make catalyst ink, 5 mg of the catalysts was dispersed in 5 mL of a mixed solvent and sonicated for 20 min. The solvent contained a mixture of de-ionized water, isopropanol, and 5% Nafion 117 solution at the

volumetric ratio of 8:2:0.05. The catalyst ink including 3 μg of Pt was added onto the RDE and dried under the air flow for 30 min to make the working electrode. The loading amount of $Pd_3Pb/Pt_nPb/C$, $Pt_xPb/PtPb/C$, PtPb/C or Pt/C catalysts on the RDE was determined to be ~15 $\mu g_{Pt}/cm^2$. For Pd_3Pb/C catalysts, the data was identical, but normalized by the amount of Pd. The electrochemical active surface area (ECSA) was determined by integrating the carbon monoxide oxidation charge via CO stripping measurements in 0.5 M H_2SO_4. The CO stripping measurements were conducted in CO-saturated electrolyte at a scan rate of 50 mV/s in the range of 0-1 V versus RHE. The cyclic voltammetry (CV) was first conducted at room temperature in Ar-purged 1 M KOH solutions at a sweep rate of 50 mV/s between 0 and 1.0 V versus reversible hydrogen electrode (RHE) for 50 cycles to clean the surface of the catalysts. Methanol oxidation reaction (MOR) was conducted in a mixture solution containing 1 M MeOH and 1 M KOH at a scan rate of 50 mV/s between 0.25 V and 1.2 V. The chronoamperometry (I-t) curves were measured at 0.77 V versus RHE for 10000 s.

Computational Method.

The adsorption energies of CO were performed by using the Vienna Ab-initio Simulation Package (VASP),[2,3] employing the density functional theory (DFT) and the Projected Augmented Wave (PAW) method.[4] The Perdew-Burke-Ernzerhof (PBE) functional was used to describe the exchange and correlation effect.[5] For all the geometry optimizations, the cutoff energy was set to be 500 eV. The surfaces of (111), (0001), and (111) were used to represent the catalytic surface of Pt, PtPb, and Pt_3Pb, respectively. The Monkhorst-Pack grids were set to be 3×3×1 for performing the surface calculations.[6] At least 16 Å vacuum layer was applied in z-direction of the slab models, preventing the vertical interactions between slabs.

The adsorption energy of CO was defined as

$$\Delta E_{ads} = E_{ads} - E_{slab} - E_{CO}$$

where E_{ads} is the electronic energy of the slab with an adsorbed CO molecule, E_{slab} is the electronic energy of the clean surface, and E_{CO} is the electronic energy of gaseous molecule. Under this definition, a more negative value indicates a stronger binding system.

Supplemental data

Supplemental tables.

Table S1. ICP-AES data of $Pd_3Pb/Pt_{3.50}Pb$, $Pd_3Pb/Pt_{2.37}Pb$, $Pd_3Pb/Pt_{2.07}Pb$ and Pd_3Pb.

Samples	Atomic ratio of Pd %	Atomic ratio of Pt %	Atomic ratio of Pb %	Pt/Pb*
$Pd_3Pb/Pt_{3.50}Pb$	16.2	61.0	22.8	3.50
$Pd_3Pb/Pt_{2.37}Pb$	14.7	56.6	28.7	2.37
$Pd_3Pb/Pt_{2.07}Pb$	14.0	54.8	31.2	2.07
Pd_3Pb	75.1	24.9	/	/
$Pt_xPb/PtPb$	/	62.1	37.9	1.64
PtPb	/	49.3	50.7	0.97

* Pt/Pb in three Pd_3Pb/Pt_nPb samples is defined as the atomic ratio of Pt and Pb after subtracting the amount of Pb in the Pd_3Pb with a Pd/Pb atomic ratio of 3:1.

Table S2. Summarized XPS data of $Pd_3Pb/Pt_{3.50}Pb$, $Pd_3Pb/Pt_{2.37}Pb$, $Pd_3Pb/Pt_{2.07}Pb$, Pt bulk, Pb bulk, and commercial Pt/C.

Samples	Pt^0 $4f_{7/2}$	Pt^0 $4f_{5/2}$	Pb^0 $4f_{7/2}$	Pb^0 $4f_{5/2}$	Pt/Pb atomic ratio
$Pd_3Pb/Pt_{3.50}Pb$	71.09 eV	74.42 eV	137.18 eV	142.04 eV	3.08
$Pd_3Pb/Pt_{2.37}Pb$	70.96 eV	74.29 eV	137.10 eV	141.96 eV	1.94
$Pd_3Pb/Pt_{2.07}Pb$	70.91 eV	74.24 eV	136.94 eV	141.80 eV	1.75
$Pt_xPb/PtPb$	71.07 eV	74.40 eV	136.69 eV	141.55 eV	1.35
PtPb	71.16 eV	74.49 eV	136.95 eV	141.81 eV	1.03
Pt bulk	71.20 eV	74.53 eV	/	/	/
Pb bulk	/	/	136.90 eV	141.76 eV	/
Pt/C	71.23 eV	74.56 eV	/	/	/

Table S3. Comparison of MOR performance of $Pd_3Pb/Pt_{2.37}Pb$ in this work with state of art electrocatalysts in the literatures.

Samples	Condition	i_m (A/mg_{Pt})	i_s (mA/cm^2)	Ref.
**$Pd_3Pb/Pt_{2.37}Pb$**	1M KOH+ 1M MeOH	8.40	13.68	This work
**PtCu Nanoframes**	0.5 M KOH+ 1M MeOH	2.55	18.2	7
**$Pt/Ni(OH)_2/rGO-4$**	1M KOH+ 1 M MeOH	1.24	1.94	8
**$Co-Pt_3$ Films**	1 M NaOH + 1 M MeOH	/	4.8	9
**PtCu–O EONDs**	0.5 M KOH+ 1 M MeOH	4.43	15.1	10
**PtZn**	0.1 M KOH+ 0.5 M MeOH	0.55	1.14	11
**Pd@PtNi NSs**	1 M NaOH+ 1 M MeOH	1.6	2.68	12
**PtAuRu/ RGO/Gc**	1 M KOH+ 1M MeOH	1.6	/	13
**SANi-PtNWs**	1M KOH+ 1 M MeOH	7.93	/	14
**Porous Pt NTs**	1 M NaOH + 1 M methanol	2.3	4.9	15
**AgAu@Pt nanoframes**	0.2M KOH+ 1 M MeOH	0.48	1.96	16
**Pt_1Ni_1/C**	1 M KOH+ 1 M MeOH	~1.7	4.9	17

Table S4. Summarized E_{ads}, E_{slab}, E_{CO}, and ΔE_{ads} for three kinds of surfaces including *fcc*-structured Pt(111), *fcc*-structured Pt_xPb(111), and hexagonal-structured PtPb(0001) obtained by DFT calculations.

Samples	E_{ads} (eV)	E_{slab} (eV)	E_{CO} (eV)	ΔE_{ads} (eV)
Pt(111)	-387.33	-370.71	-14.77	-1.85
Pd_xPb(111)	-356.24	-339.76	-14.77	-1.71
PtPb(0001)	-301.71	-285.34	-14.77	-1.60

Supplemental figures.

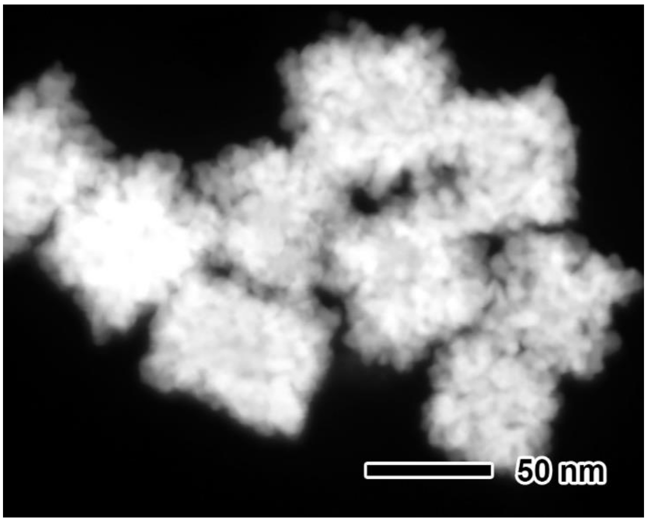

Figure S1. HAADF-STEM image of the $Pd_3Pb/Pt_{2.37}Pb$ nanocubes prepared using the standard procedure.

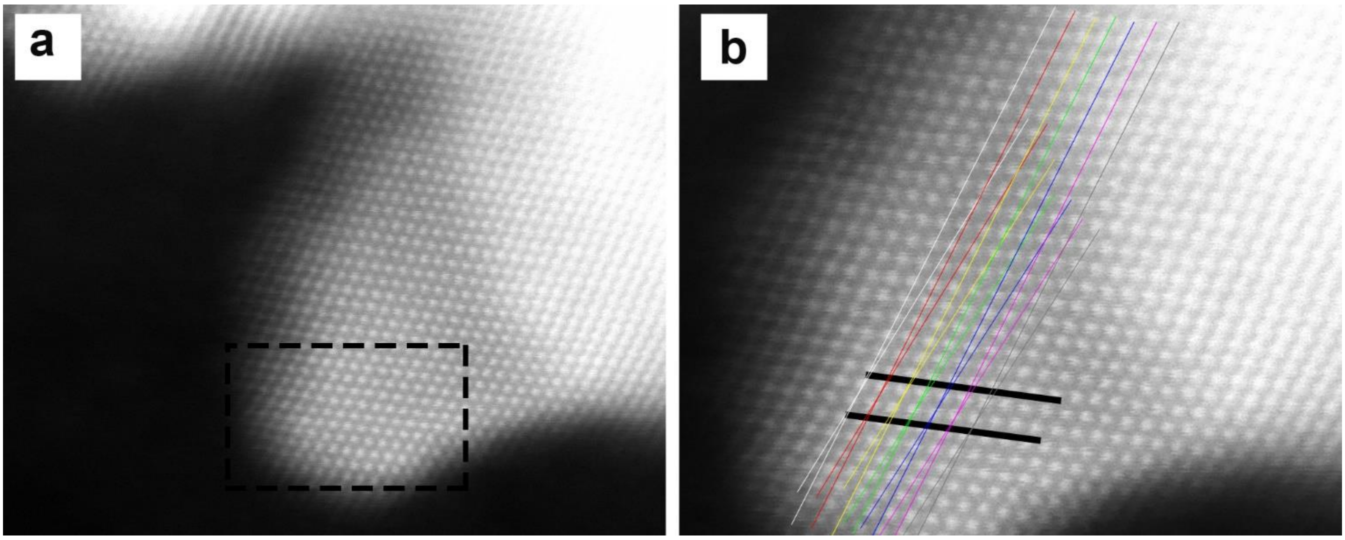

Figure S2. (a, b) Atomic-resolution HAADF-STEM images of the tip of the $Pd_3Pb/Pt_{2.37}Pb$ cubes. In (b), we selected several atoms in the top and drew a line. From the down, another homochromatic line was drawn along the atoms. These two lines intersected at the interface. Repeat this operation and the phase interface was obtained, which was marked by two black parallel lines.

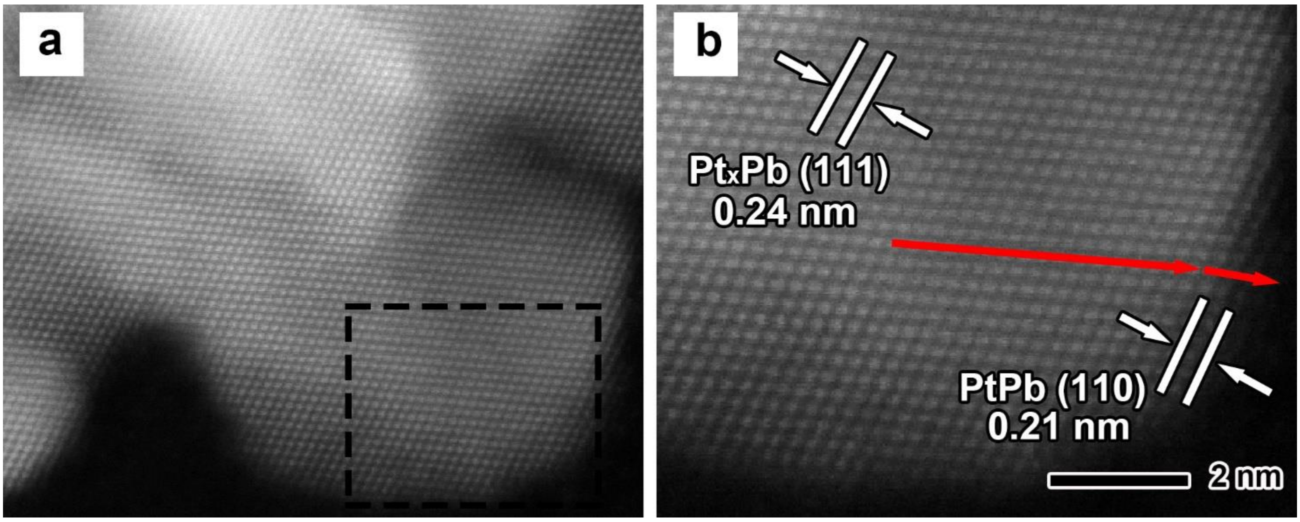

Figure S3. (a, b) Additional HAADF-STEM images of $Pd_3Pb/Pt_{2.37}Pb$ cubes in another region.

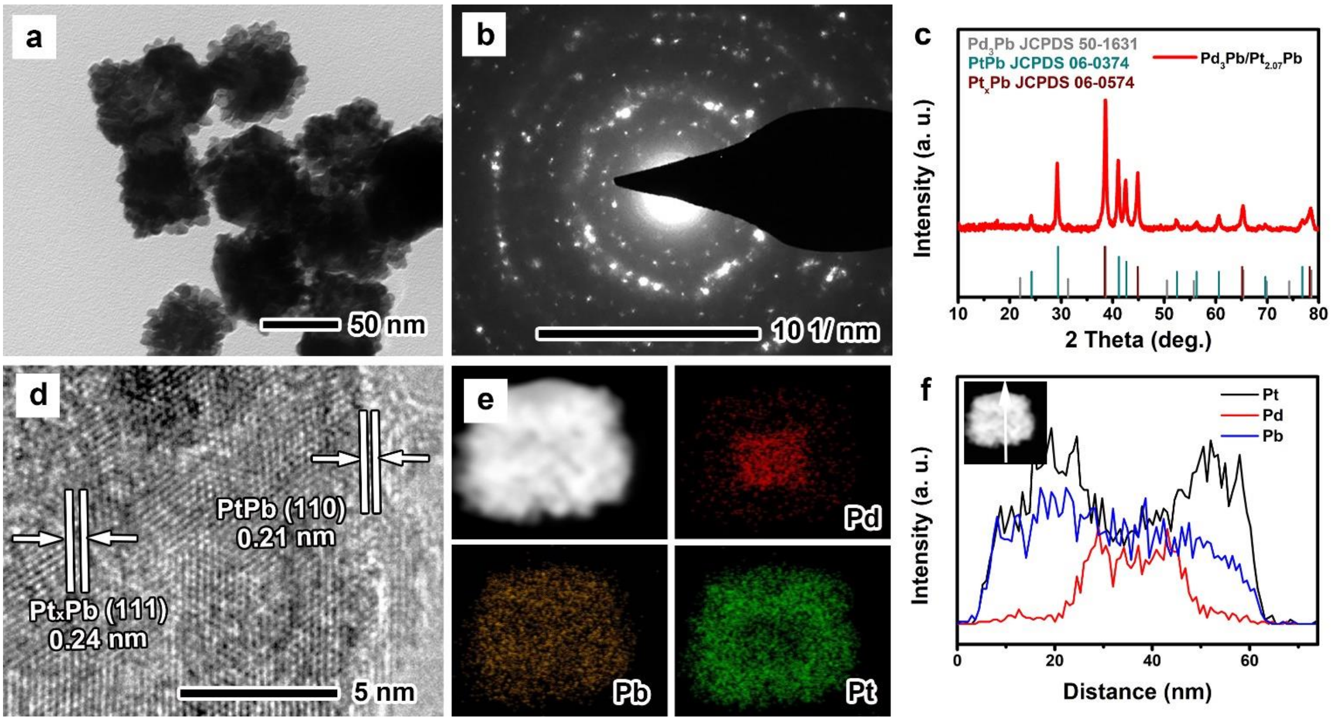

Figure S4. (a) TEM image, (b) SAED pattern, (c) XRD pattern, (d) HRTEM image, (e) EDX mapping image and (f) line-scan profiles of the $Pd_3Pb/Pt_{2.07}Pb$ nanocube prepared by adding 12 mg of $Pb(acac)_2$.

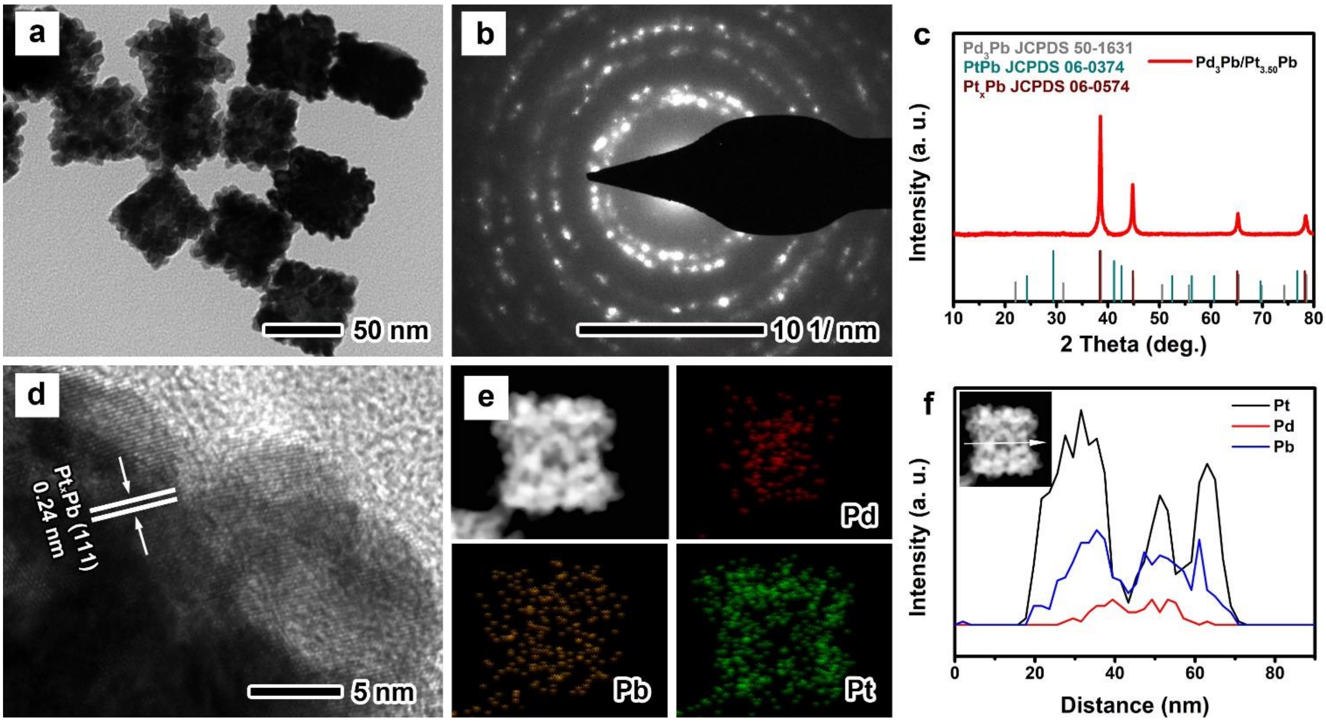

Figure S5. (a) TEM image, (b) SAED pattern, (c) XRD pattern, (d) HRTEM image, (e) EDX mapping image and (f) line-scan profiles of the $Pd_3Pb/Pt_{3.50}Pb$ nanocube prepared by adding 7 mg of $Pb(acac)_2$.

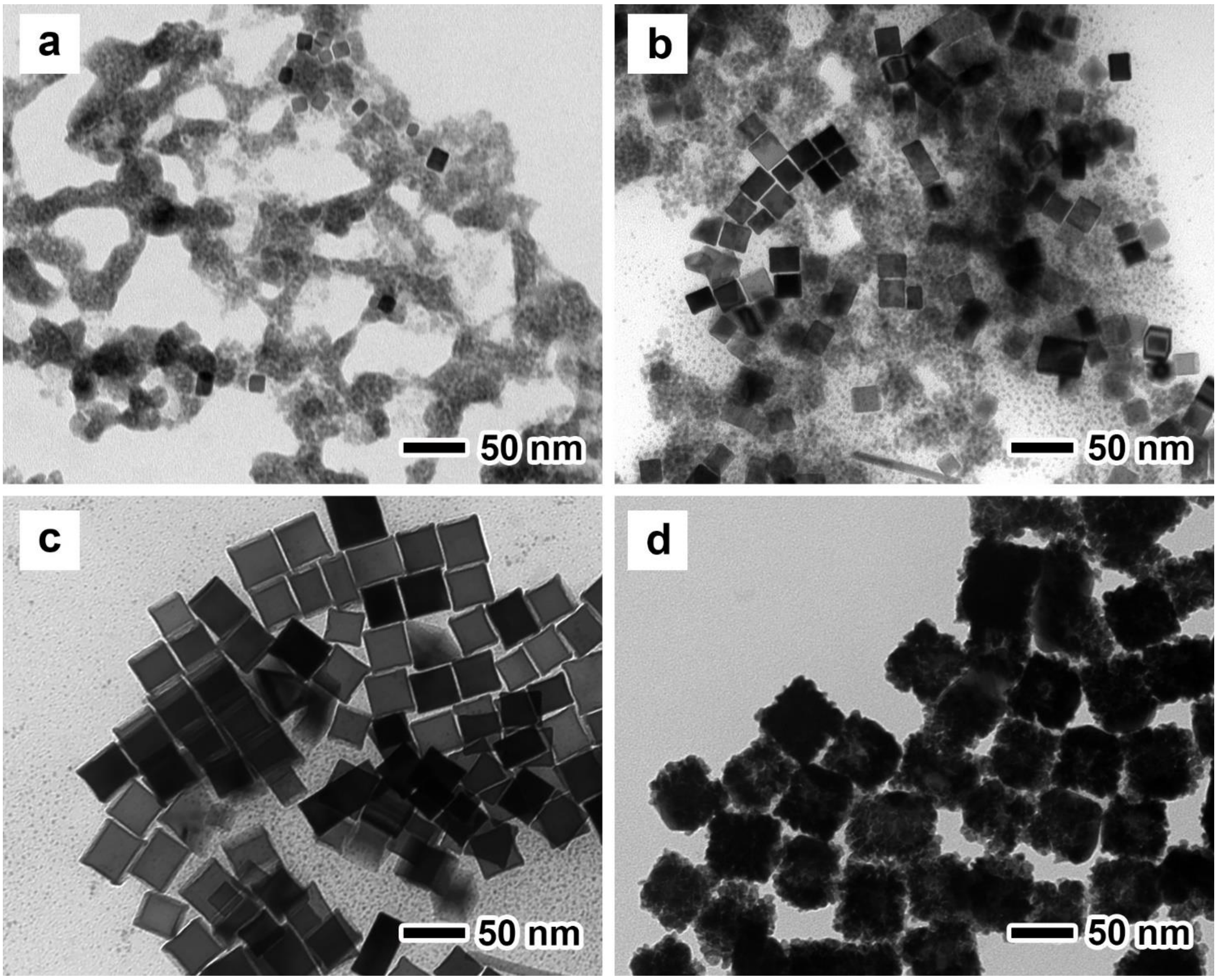

Figure S6. TEM images of the $Pd_3Pb/Pt_{2.37}Pb$ prepared using the standard procedure at different reaction times: (a) 10, (b) 15, (c) 20, and (d) 30 min.

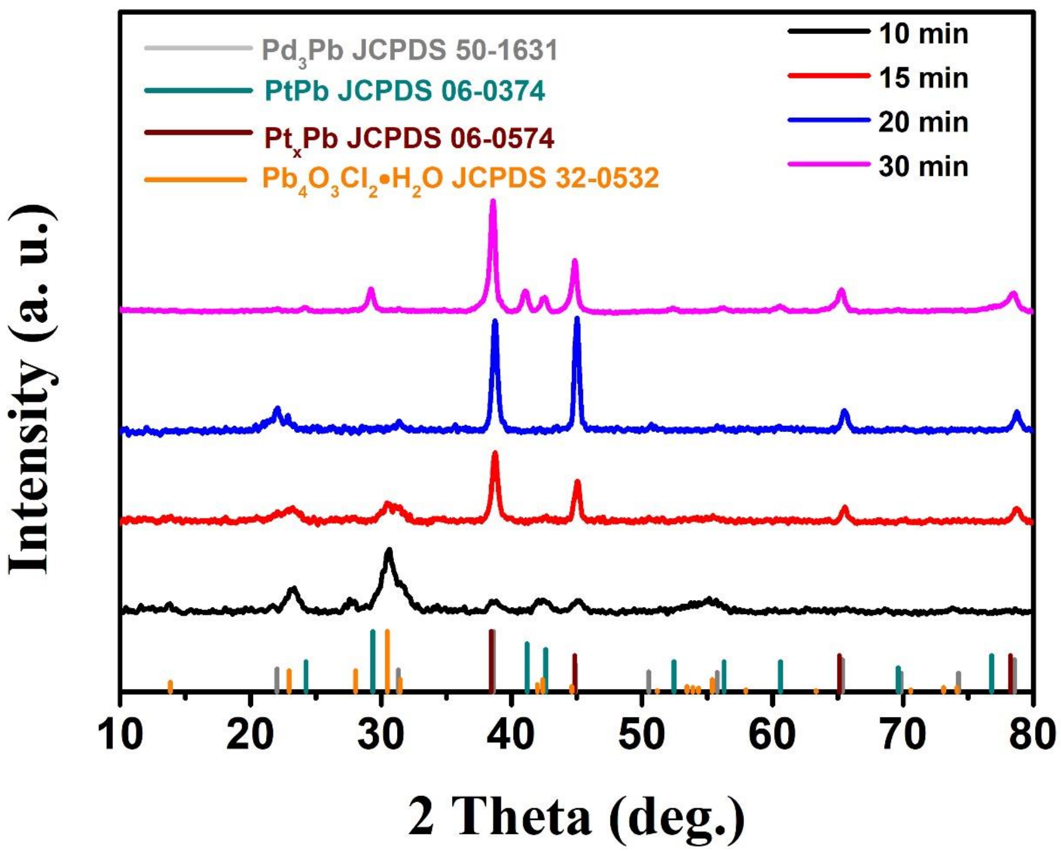

Figure S7. XRD patterns of the $Pd_3Pb/Pt_{2.37}Pb$ prepared using the standard procedure at different reaction times: (a) 10, (b) 15, (c) 20, and (d) 30 min.

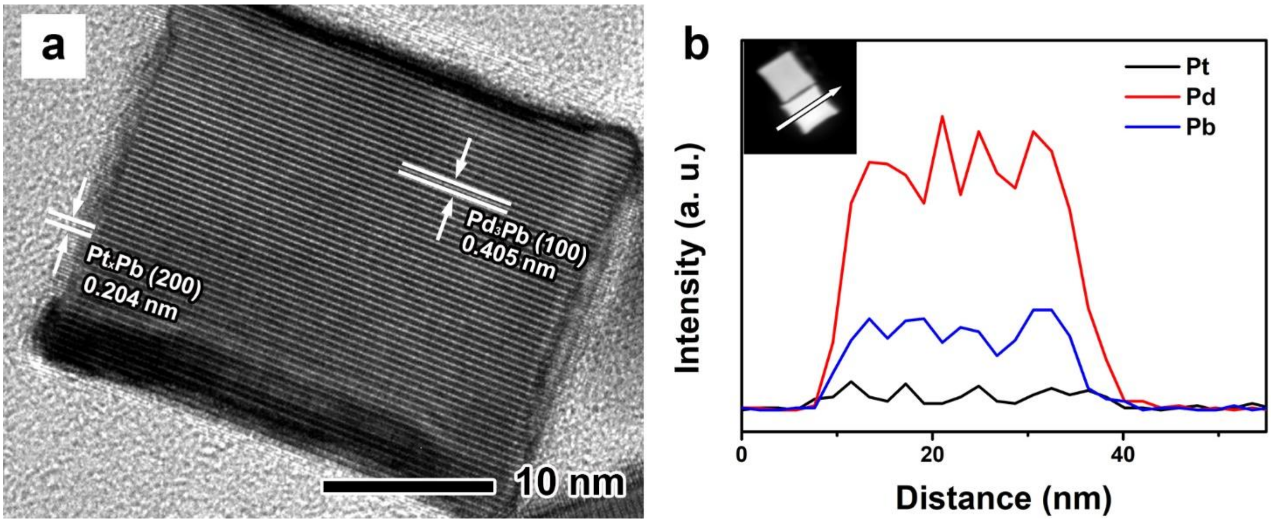

Figure S8. (a) HRTEM image (b) EDX line-scan spectra of the product prepared at a reaction time of 20 min.

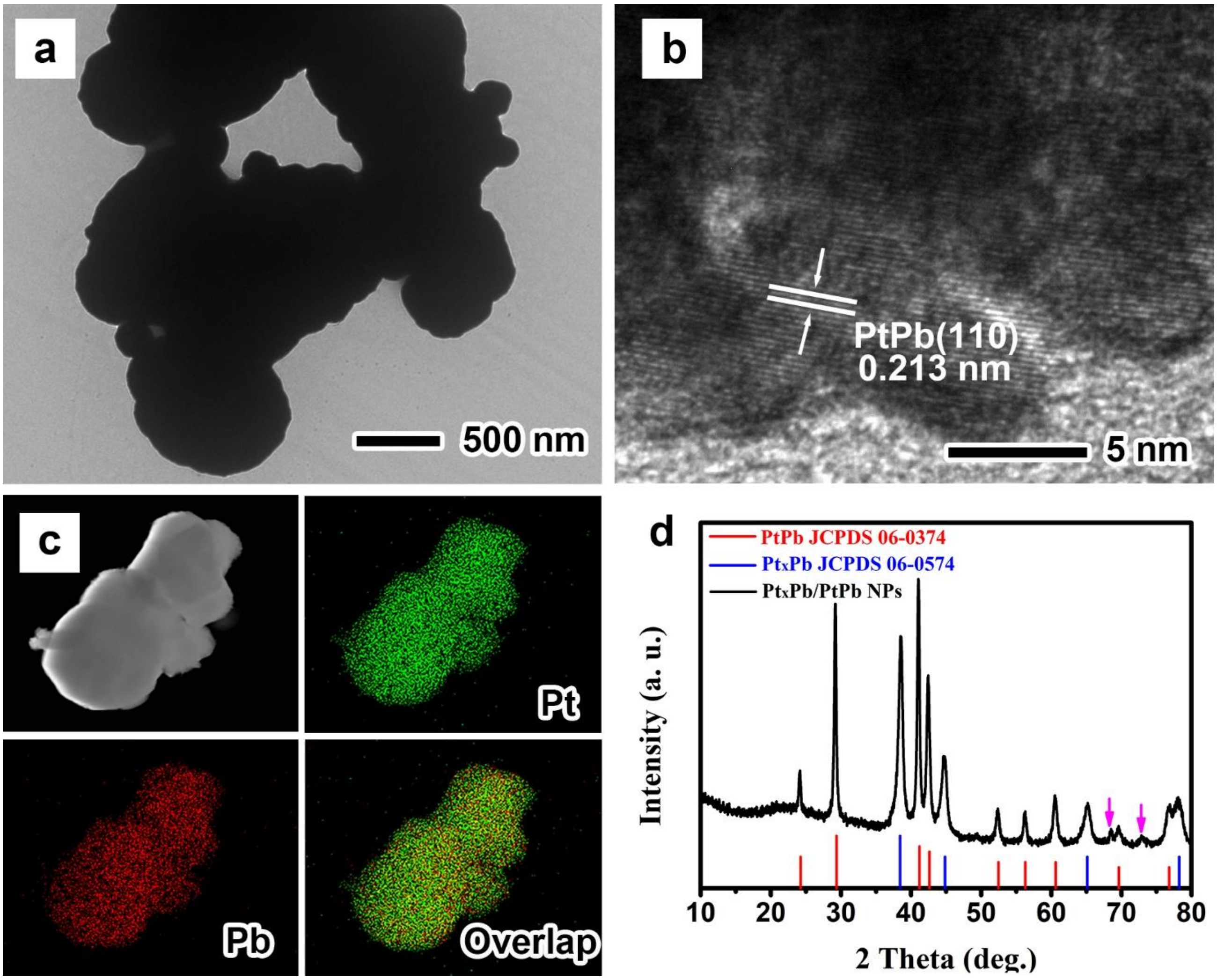

Figure S9. (a) TEM image, (b) HRTEM image, (c) EDX mapping image and (d) XRD pattern of the Pt_xPb/PtPb particles that obtained the absence of $Pd(acac)_2$ with other conditions being the same as the standard procedure.

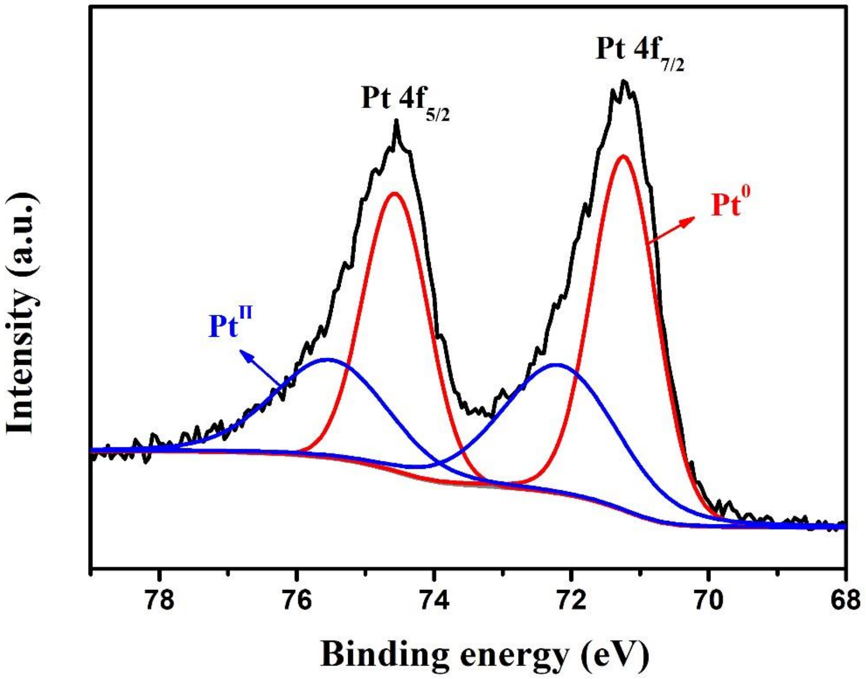

Figure S10. Pt 4f XPS spectra of commercial Pt/C.

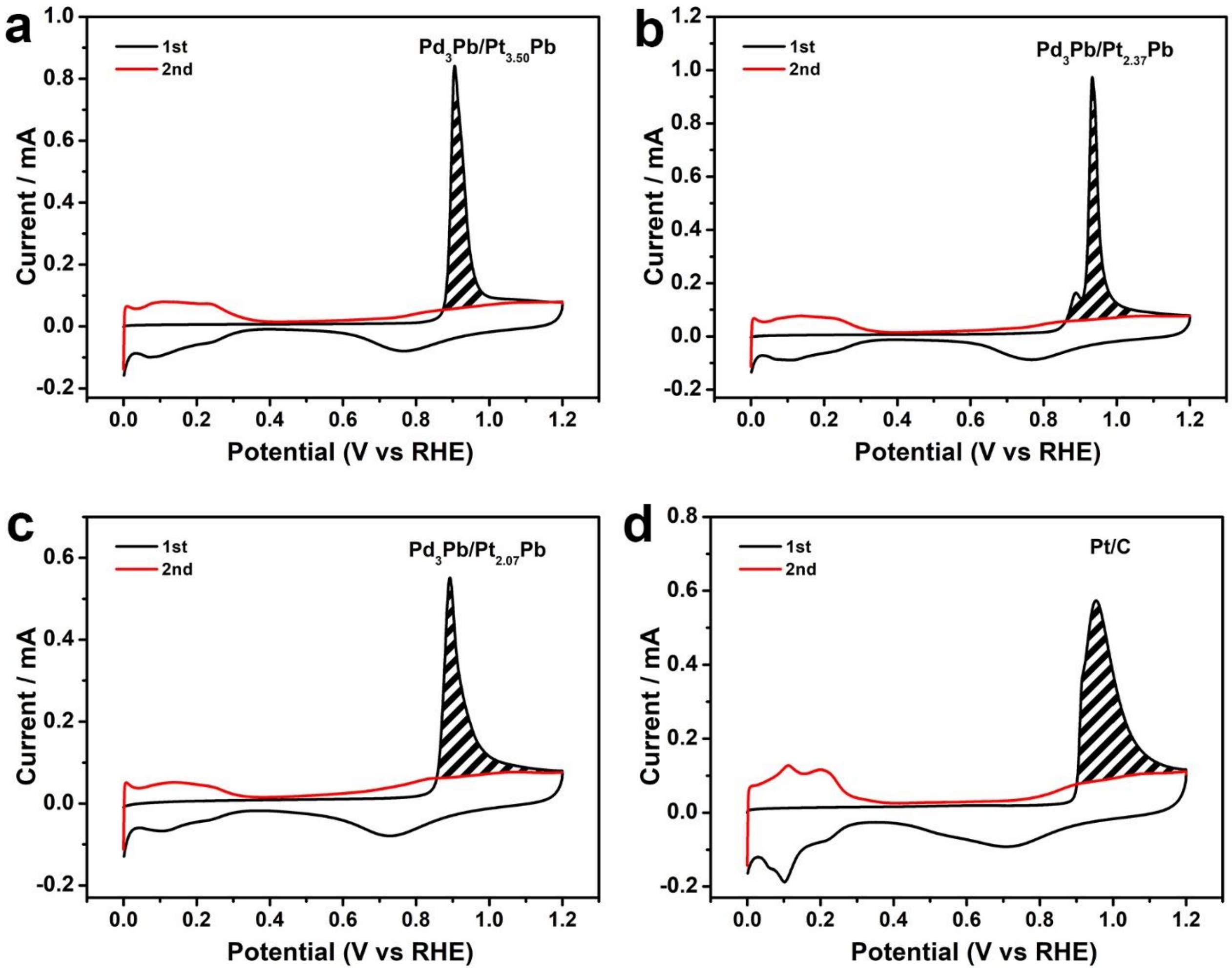

Figure S11. CO stripping curves of four catalysts recorded in 0.5 M H_2SO_4 solution at a sweep rate of 50 mV/s. The area of the shaded area represents the ECSAs.

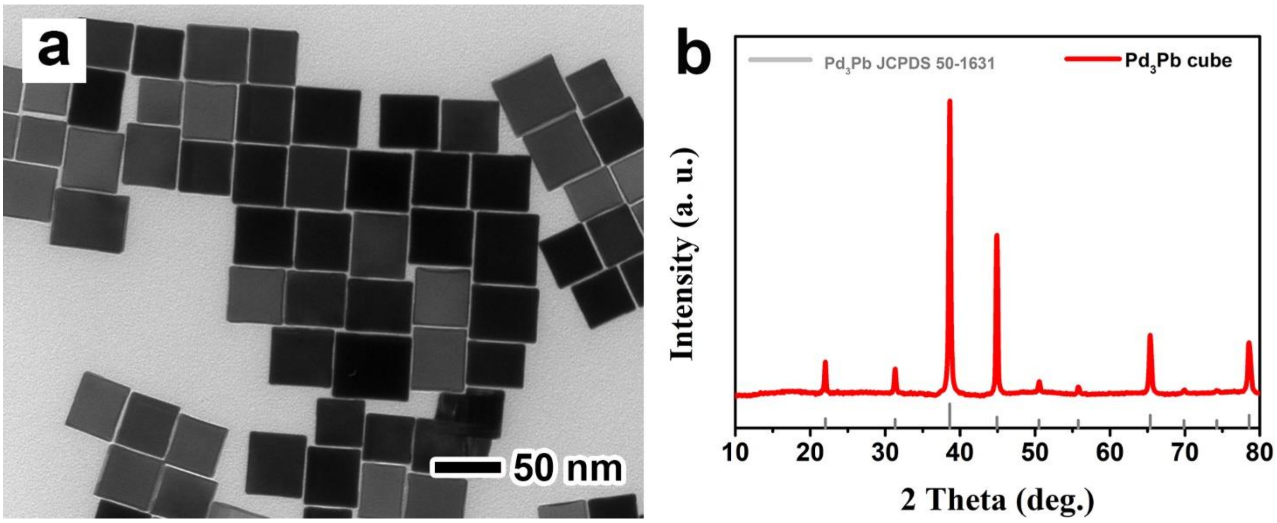

Figure S12. (a) TEM image and (b) XRD pattern of the Pd_3Pb nanocubes.

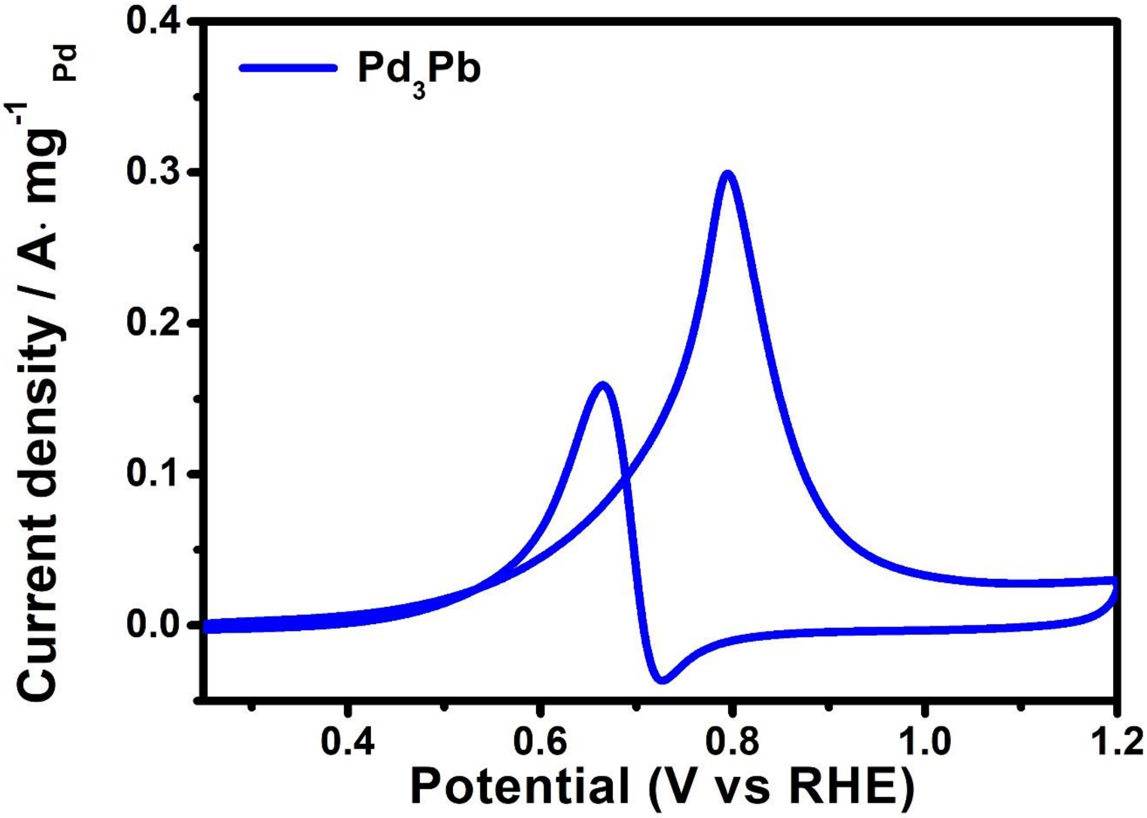

Figure S13. CV curves of the Pd_3Pb/C measured in a mixed solution containing 1 M KOH and 1 M MeOH with a scan rate of 50 mV/s at room temperature normalized by Pd mass.

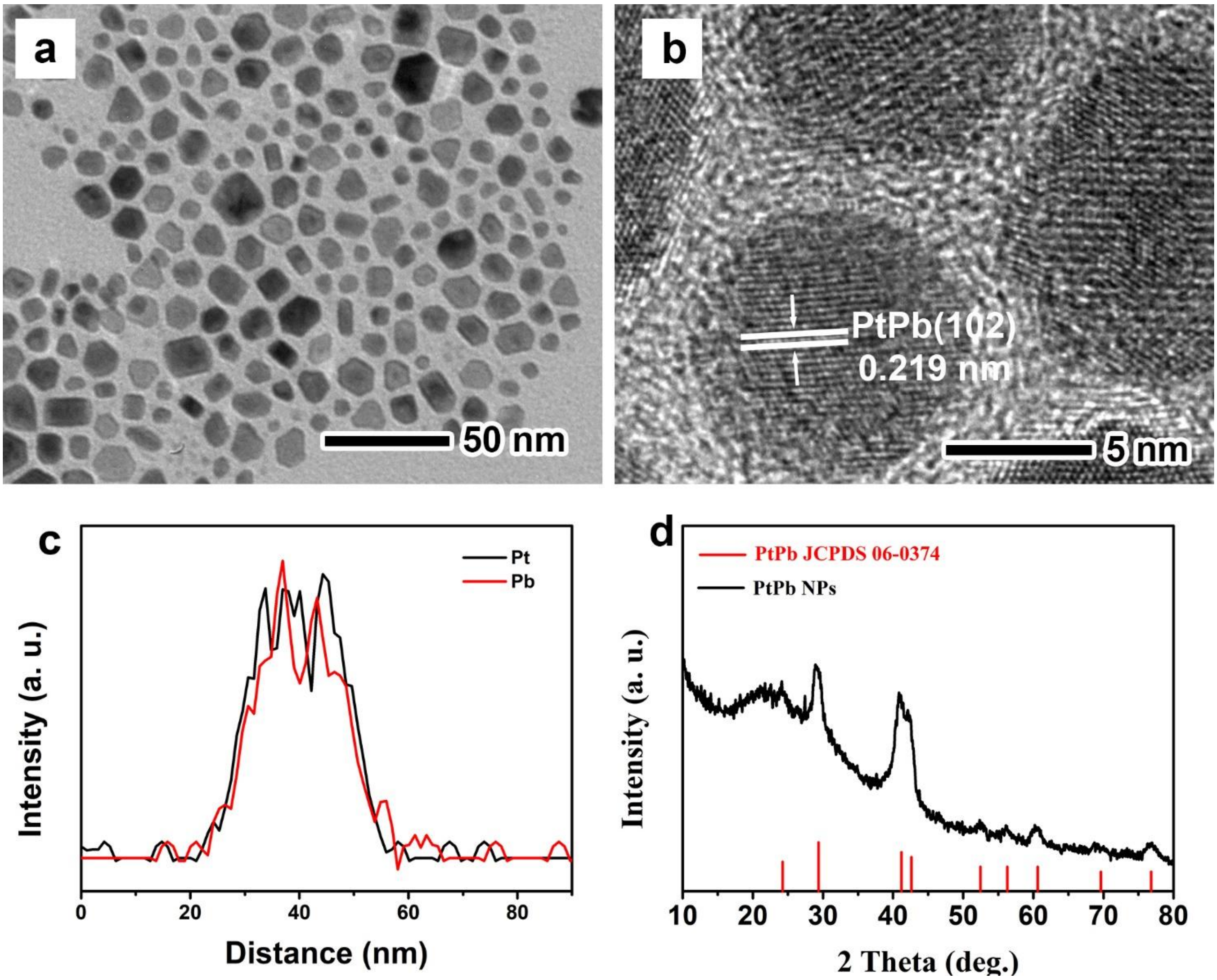

Figure S14. (a) TEM image, (b) HRTEM image, (c) EDX line-scan spectra and (d) XRD pattern of the PtPb nanoparticles.

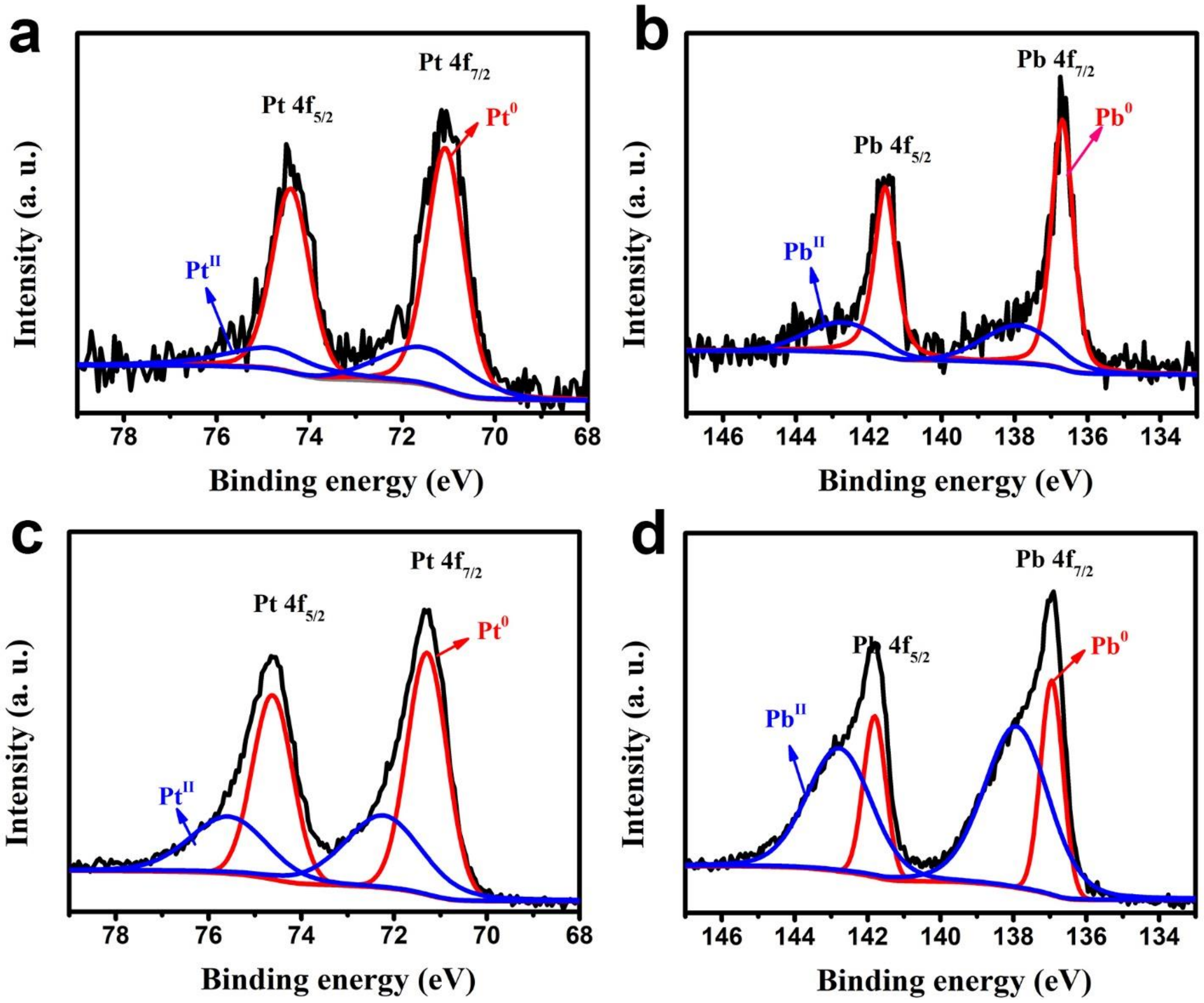

Figure S15. (a, c) Pt 4f and (b, d) Pb 4f XPS spectra of the Pt_xPb/PtPb and PtPb nanoparticles, respectively.

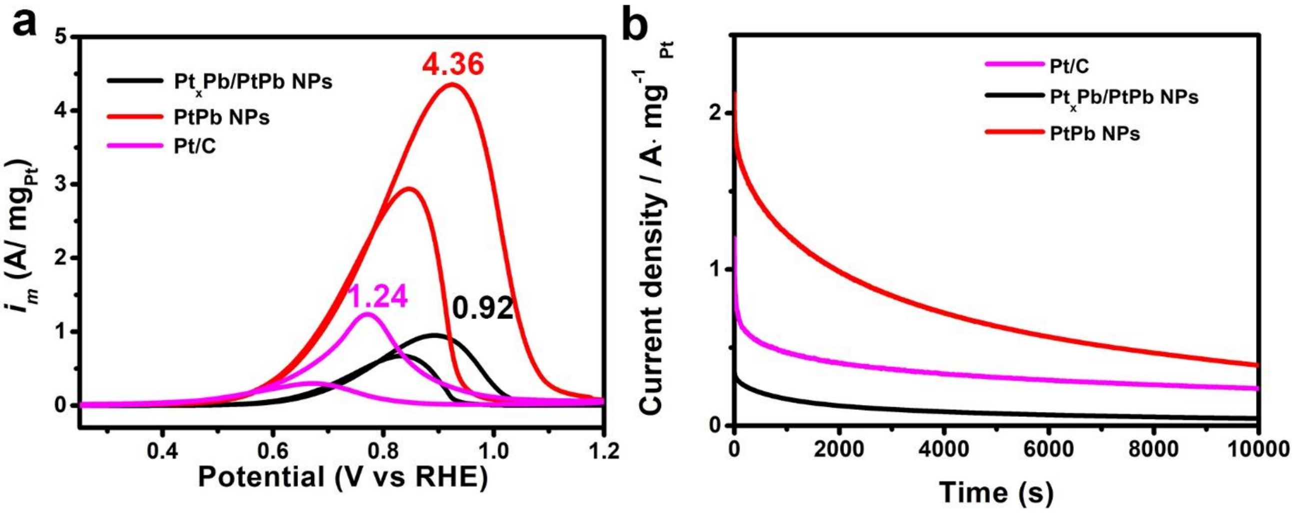

Figure S16. (a) Cyclic voltammograms (*CV*) of the Pt_xPb/PtPb, PtPb nanoparticles and commercial Pt/C in a mixed solution containing 1 M KOH and 1 M MeOH at a scan rate of 50 mV/s for MOR normalized by Pt mass. (b) Current−time (*I*−*t*) curves for MOR at 0.77 V (vs RHE) for 10000 s.

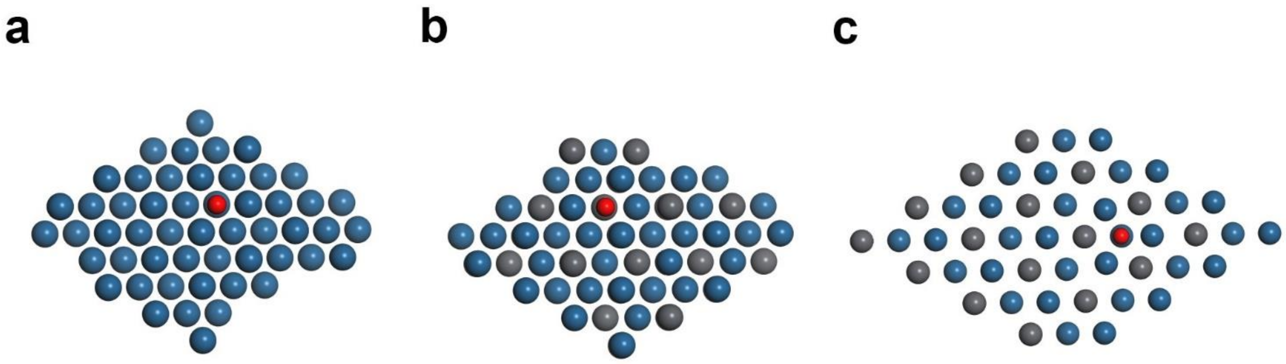

Figure S17. The top view of structure models corresponding to (a) Pt(111), (b) $Pt_xPb(111)$, and (c) PtPb(0001).

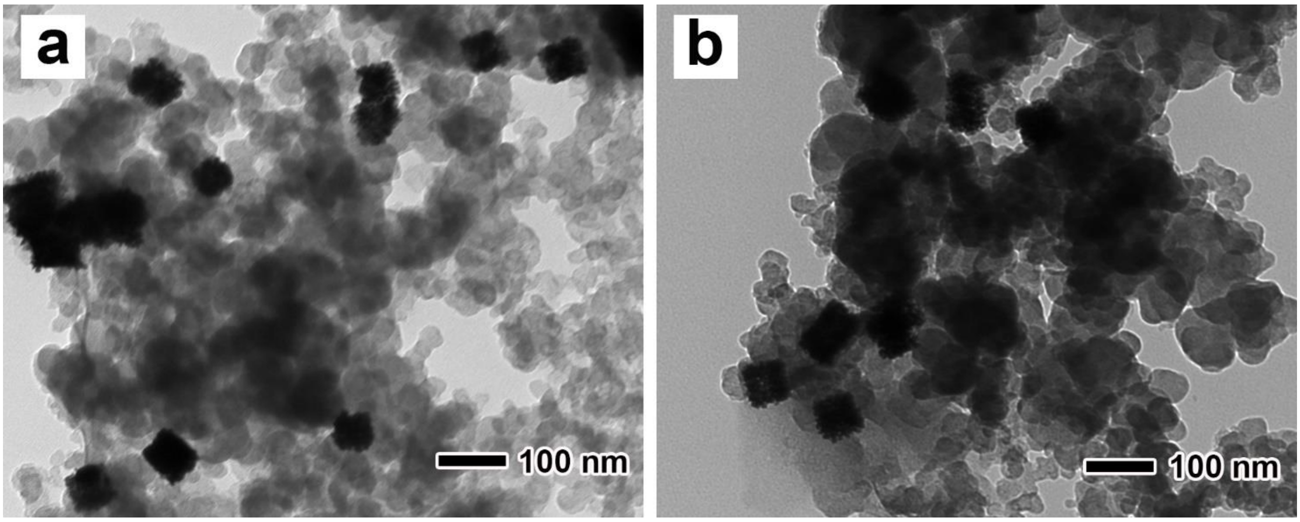

Figure S18. TEM images of the $Pd_3Pb/Pt_{2.37}Pb$ nanocubes that loaded on the carbon support (a) before and (b) after 10000 s stability test.

References

[1] B. E, Q. Shao, L. Bu, S. Bai, Y. Li, X. Huang, *Adv. Energy Mater.* **2018**, *8*, 1703430.

[2] G. Kresse and J. Furthmüller, *Phys, Rev. B* **1996**, *54*, 11169-11186.

[3] G. Kresse and J. Hafner, *Phys. Rev. B* **1994**, *49* 14251-14269.

[4] P.Blöchl, *Phys. Rev. B* **1994**, *50*, 17953-17979.

[5] J. Perdew, K. Burke and M. Ernzerhof, *Phys. Rev. Lett*. **1996**, *77*, 3865-3868.

[6] H. Monkhorst and J. Pack, *Phys, Rev. B* **1976,** *13*, 5188.

[7] Z. Zhang, Z. Luo, B. Chen, C. Wei, J. Zhao, J. Chen, X. Zhang, Z. Lai Z. Fan, C. Tan, M. Zhao, Q. Lu, B. Li, Y. Zong, C. Yan, G. Wang, Z. Xu, and H. Zhang, *Adv. Mater.* **2016**, *28*, 8712-8717.

[8] W. Huang, H. Wang, J. Zhou, J. Wang, P. Duchesne, D. Muir, P. Zhang, N. Han, F. Zhao, M. Zeng, J. Zhong, C. Jin, Y. Li, S. Lee and H. Dai, *Nat. Commun.* **2015**, *6*, 10035.

[9] A. Serrà, E. Gómez, I. Golosovsky, J. Nogués and E. Vallés, *J. Mater. Chem. A* **2016**, *4*, 7805-7814.

[10] F. Wu, L. Zhang, J. Lai, W. Niu, R. Luque, and G. Xu, *J. Mater. Chem. A* **2019**, *7*, 8568-8572.

[11] Z. Qi, C. Xiao, C. Liu, T. Goh, L. Zhou, R. Maligal-Ganesh, Y. Pei, X. Li, L. Curtiss, and W. Huang, *J. Am. Chem. Soc.* **2017**, *139*, 4762-4768.

[12] G. Ren, Y. Liu, W. Wang, M. Wang, Z. Zhang, Y. Liang, S. Wu and J. Shen, *ACS Appl. Nano Mater.* **2018**, *1*, 3226-3235.

[13] F. Ren, C. Wang, C. Zhai, F. Jiang, R. Yue, Y. Du, P. Yang and J. Xu, *J. Mater. Chem. A* **2013**, *1*, 7255.

[14] M. Li, K. Duanmu, C. Wan, T. Cheng, L. Zhang, S. Dai, W. Chen, Z. Zhao, P. Li, H. Fei, Y. Zhu, R. Yu, J.

Luo, K. Zang, Z. Lin, M. Ding, J. Huang, H. Sun, J. Guo, X. Pan, W. Goddard III, P. Sautet, Y. Huang and X. Duan, *Nat. Catal.* **2019**, *2,* 495-503.

[15] Y. Lou, C. Li, X. Gao, T. Bai, C. Chen, H. Huang, C. Liang, Z. Shi and S. Feng, *ACS Appl. Mater. Interfaces* **2016**, *8*, 16147-16153.

[16] X. Yan, S. Yu, Y. Tang, D. Sun, L. Xu and C. Xue, *Nanoscale* **2018**, *10*, 2231-2235.

[17] S. Lu, H. Li, J. Sun and Z. Zhuang, *Nano Res.* **2018**, *11*, 2058.
